# The legacy of large dams in the United States

**DOI:** 10.1007/s13280-021-01533-x

**Published:** 2021-03-08

**Authors:** Giuliano Di Baldassarre, Maurizio Mazzoleni, Maria Rusca

**Affiliations:** 1 Department of Earth Sciences, Uppsala University, Uppsala, Sweden; 2 Centre of Natural Hazards and Disaster Science, CNDS, Uppsala, Sweden; 3 Department of Integrated Water Systems and Governance, IHE Delft, Delft, The Netherlands

**Keywords:** Agricultural expansion, Droughts, Population growth, Sustainability, Water crisis, Water infrastructure

## Abstract

The sustainability of large dams has been questioned on several grounds. One aspect that has been less explored is that the development of dams and reservoirs often enables agricultural expansion and urban growth, which in turn increase water consumption. As such, dam development influences, while being influenced by, the spatial and temporal distribution of both supply and demand of water resources. In this paper, we explore the interplay between large dams, patterns of population growth and agricultural expansion in the United States over the past two centuries. Based on a large-scale analysis of spatial and temporal trends, we identify three distinct phases, in which different processes dominated the interplay. Then, we focus on agricultural water use in the Southwest region (Arizona, California and Nevada) and explore chicken-and-egg dynamics where water supply partly meets and partly fuels water demand. Lastly, we show that the legacy of dams in the United States consists of a lock-in condition characterized by high levels of water consumption, especially in the Southwest, which leads to severe water crises and groundwater overexploitation when droughts occur.

## Introduction

For most of the 20th century, large dams represented a core strategy to promote economic growth. Between 1950 and 2000, the number of large dams increased from 5000, mostly located in industrialized countries, to over 45,000 worldwide (Richter et al. 2010). Dam developments slowed down at the turn of the century, following increased protests in the 1990s and the publication of the report by the World Commissions on Dams (WCD Secretariat 2000), which claimed that their social and environmental costs outweighed their benefits. In recent years, however, a new wave of large dam development has been witnessed globally (Zarfl et al. 2015; Crow-Miller et al. 2017; Latrubesse et al. 2017; Rusca et al. 2019). Many of these new projects revolve around the promise of clean energy production and the need to secure water supply (Briscoe 2009; Hirsch 2010; Schulz and Adams 2019).

Several scholars have questioned the sustainability of dams and reservoirs because of their environmental and social impacts (Rufin et al. 2019), which include negative implications for human health (Keiser et al. 2005) and livelihoods (Kirchherr et al. 2016), as well as alteration of hydrological regimes (Jaramillo and Destouni 2015) that severely affect biodiversity (Barbarossa et al. 2020) and ecological processes (Santos et al. 2020).

One less explored issue with reservoirs is that by supplying more water, food and energy, they enable agricultural, urban and industrial expansion that, in turn, lead to growing demands (Kallis 2010; Gohari et al. 2013; Di Baldassarre et al. 2018). This feedback can quickly offset the original benefits of reservoirs. For instance, new reservoirs that secure water supply for irrigation increase the profitability of crop production, thereby triggering agricultural expansion and increasing water consumptions (Thomas and Adams 1999; Rufin et al. 2019). These growing demands can then set in motion further reservoir expansion or construction of additional dams (Gohari et al. 2013). This phenomenon has been described in the recent literature as supply-demand cycle (Kallis 2010; Di Baldassarre et al. 2018). Supply-demand cycles can generate accelerating spirals towards unsustainable water consumption (Di Baldassarre et al. 2018), environmental degradation (Kallis 2010) and peak water limits (Gleick and Palaniappan 2010).

The interplay between supply and demand of water resources has been analysed for specific case studies at the local scale (Kallis 2010; Gohari et al. 2013; Di Baldassarre et al. 2018), but it remains largely unknown how dam developments enable supply-demand cycles and propagate in space and time across multiple scales.

In this paper, we examine the legacy of dam development in the United States, by exploring how changes (in time and space) in human population, water supply and agricultural expansion have shaped each other over the past two centuries. To this end, we draw from methods and concepts of the interplay of human and water systems that have been recently developed in environmental history and political ecology (Scott 1999; Kaika 2006; Molle et al. 2009; Wester 2009; Rusca et al. 2019) as well as social-ecological systems and sociohydrology (Sivapalan et al. 2012; Di Baldassarre et al. 2015; Pan et al. 2018, 2020; Hall 2019; Kalantari et al. 2019).

## Case study

### Geographical context

**Fig. 1:**
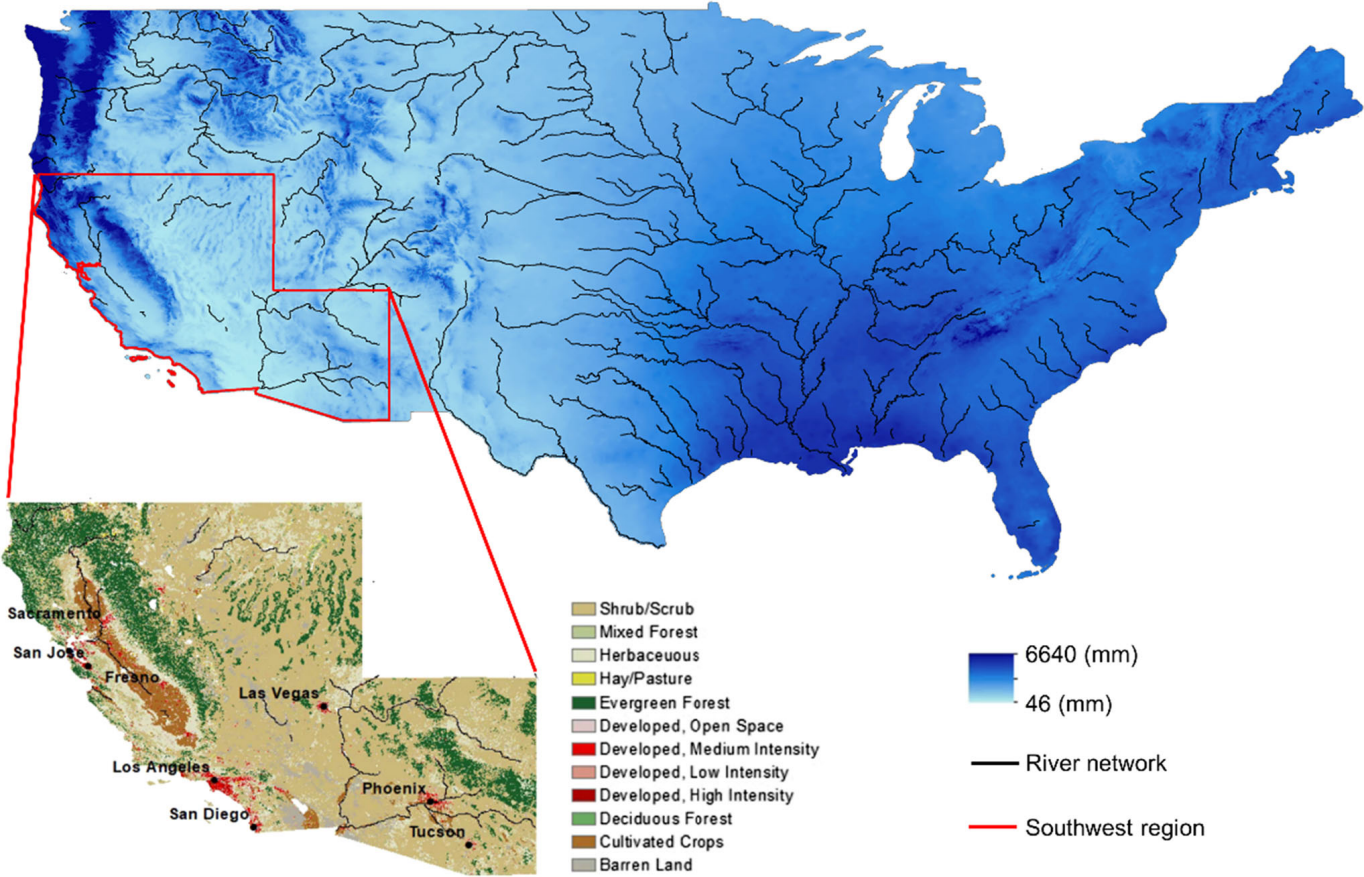
Study area. Map of the US showing the spatial variability of mean annual precipitation between 1981 and 2010 (PRISM Climate Group 2004), and zoom inset on the Southwest region (Arizona, California and Nevada) highlighting the high presence of developed, urbanized and agricultural areas in a relatively dry area (National Land Cover Database for the conterminous US for 2011 reported in Homer et al. 2015)

The contiguous United States (hereafter US) was selected as an instrumental case study to explore the legacy of dam developments for various reasons. First, the US is exposed to multiple hydroclimatic conditions, and it includes both wet and dry regions as depicted in Fig. 1. Second, there is broad availability of data in the US, which also allows going across different temporal and spatial scales. Hence, our results about the sustainability of large dams can provide useful insights for similar developments in other places around the world.

Water resources played a key role in the establishment and development of the US and, in turn, the economic, socio-political, environmental and hydrological consequences of transforming rivers and diverting watercourses shaped contemporary American society. The guiding principles and logics of dam developments changed over the past two centuries, because of historical and geopolitical developments, as well as contextual factors. Concurrently, hydrologic and climatic conditions co-shaped dam developments, patterns of human population and water consumptions as described in the following section.

### Historical background

In the 19th century, dam development in the US was mostly focused on navigation (Billington et al. 2005). Steamboats and canals were to provide long-distance and inexpensive trade-routes between city markets, such as Chicago, New Orleans, New York and Pittsburgh (Shaw 1993; Solomon 2010). Concurrently, the urban population increased from 1.1 to 6.2 million between 1830 and 1860 (Billington et al. 2005), raising significant concerns of public health. With cholera and yellow fever outbreaks occurring in many Eastern and Western cities, the development of networked water supplies became another driver for water resources development (Pisani 2003).

For most of the 20th century, water resources development in the US was informed by high‐modernist beliefs (Forest and Forest 2012) which martialized primarily in the construction of large dams to reclaim the arid West of the US. John Wesley Powell, Director of the US Geological Survey between 1881 and 1894, was a central figure in this development. Already in 1874 he warned the Congress that agriculture as developed in the East was unsustainable beyond the 99th meridian (Solomon 2010). In the Report on the Arid Land of the Arid Region of the US (1878), he claimed that in the West, encompassing 40% of the total land of the country, “the climate is so arid that agriculture is not successful without irrigation” (Powell 1879). He championed the model of the ‘Jeffersonian yeoman’, small farmers to be supported by government-led infrastructural irrigation projects.

Powell’s project was antagonized by private vested interests of land speculators, large farmers and railroad companies who encouraged faster and larger migration to the West, by proclaiming that “rain follows the plow” (Reisner 1993). Powell resigned from the Geological Survey in 1894, but his vision remained prominent in the first part of the 20th century. In 1902, President Teddy Roosvelt (1901–1909) emanated the Water Reclamation Act (1902), which incorporated several of Powell’s ideas. The Act aimed to foster development in the arid lands of the West and “lure the landless man to the manless land” (Pisani 2003). Between 1902 and 1915, the Reclamation Bureau, which was established with the 1902 Act to develop water resources project in the West, constructed 100 dams 1300 miles of canals and 25 miles of tunnels to supply water to 20 000 small farmers (Pisani 2003). Many, however, considered Powell’s vision paternalistic. Moreover, farmers in the East were increasingly concerned about the competition from the West and possible land devaluations.

Despite this, the Bureau of Reclamation started the construction of the Hoover Dam in the 1930s. After two decades of lobbying of Southern Californian urban, farming and railroad interests, the project gained momentum (Solomon 2010). The construction also aimed to create jobs to mitigate the impacts of the economic crisis of the 1930s, unify the nation after two World Wars and the incumbent Cold War, and sustain industrial production (Swatuk 2019). The Hoover Dam provides power (Solomon 2010) for private and public utilities in the Southwest region, i.e. Arizona, California and Nevada. It also serves as a water supply reservoir for Southern California and Tucson, Phoenix, and irrigates an estimated one million acres land in central Arizona.

The dam development vision of the US started shifting in the 1940s. The idea of the family farm and of a non-commercial agrarian society was increasingly questioned and, ultimately, was replaced by a market-based model. In 1945, the size of land and quantity of water per farm was doubled. Subsequent reforms slowly introduced market-based principles and incentives for large scale agroindustry (Pisani 2003). Concurrently, the bureau started focusing more on cities as their main and more profitable customers. To illustrate, the San Luis Dam (1968) was the first large infrastructure developed to exclusively serve corporate landowners such as Southern Pacific Railroad and Standard Oil Company. In the 1970s, family farms were increasingly seen as an anachronistic model and in 1982 Congress approved a law that increased the size of farms from 160 to 960 acres, formally ending the era of family farms and acknowledging the role of the market in setting the size of the farms (Reisner 1993; Worster 2002).

In the 1980s, dam development slowed down. First, ideal sites for the construction of dams had already been exploited or had become protected by conservation policies (Perry and Praskievicz 2017). Most rivers had already been dammed in multiple locations, with the risk that one collapse (e.g. Glen Canyon dam) may cause others too (e.g. Boulder and Davis dams). The failure of the Tuton dam was a clear sign that dams were now being developed in areas previously deemed unsuitable (Reisner 1993). In some cases, the Bureau provided figures that showed a more positive cost-benefit ratio to make them more attractive to the public (Pisani 2003). Slowly, the credibility of these large-scale projects and of the Bureau itself were called into question. In 1977–78, President Carter cut 19 water projects and his successor Regan further reduced financial contributions to the Bureau (Perry and Praskievicz 2017). At the end of 1980s, the Bureau formally ended dam developments in the West. Yet, the legacy of dams remains, with significant impacts in the Southwest region (Fig. 1).

This Southwest has grown through extensive infrastructure developments, aimed at meeting conflicting demands of agriculture and cities (Gleick 2018). With more than 90% of the population living in urban centers, today the Southwest region has the highest urban concentration of the country (Melillo et al. 2017). As a result, cities such as Los Angeles, Phoenix and Las Vegas have expanded in environmentally, economically, and socially unsustainable ways (Scarrow 2014). Ensuring adequate drinking water supplies has become one of the major challenges faced by these megacities (Melillo et al. 2017). Moreover, despite being the most arid and water stressed region in the United States, over the past decades the Southwest has become one of the largest agricultural producers worldwide (Cooley 2016). The region is often portrayed as a disastrous development, generated by cheap land and water subsidized by the federal government, and lenient environmental policies (Reisner 1993; Scarrow 2014; Gleick 2018).

These developments are even more concerning when considering that hydrological and meteorological extremes are likely to be exacerbated by climate change (Sprague and Prenger-Berninghoff 2019; Williams et al. 2020). The consequences of unsustainable water developments in the Southwest in conjunction with climate change are beginning to materialise. During the 2012–2016 drought, the Governor of California Brown Jr., together with the State Water Resources Control Board, had to enforce water rationing measures (Sprague and Prenger-Berninghoff 2019). A few years before, in 1999–2004, a major drought occurred in the upper Colorado river basin (Piechota et al. 2004) and affected Las Vegas (Nevada), Tucson and Phoenix (Arizona) and Los Angeles and San Diego (California).

## Methods

In this section, we first describe the methods used to assess spatial and temporal trends of dams and population in the US over the past two centuries (1840–2010). Then, we report the statistical approach implemented for exploring the interplay between agricultural water supply and use in the Southwest in the period 1950–1980.

### Large-scale analysis

We performed a macroscopic study of the spatial and temporal dynamics of dam locations and human population over the past two centuries by analyzing changes of their corresponding center of mass (CoM) across the US in the period 1840–2010. The CoM was calculated as the average position of all the components of the system, weighted based on their masses:

1 $$ {\text{CoM}}_{x} = \frac{{\sum\nolimits_{i = 1}^{N} {m_{i} \cdot x_{i} } }}{{\sum\nolimits_{i = 1}^{N} {m_{i} } }},\quad {\text{CoM}}_{y} = \frac{{\sum\nolimits_{i = 1}^{N} {m_{i} \cdot y_{i} } }}{{\sum\nolimits_{i = 1}^{N} {m_{i} } }} $$

where *N* represents the total number of components in a given dataset, *x*_*i*_ and *y*_*i*_ are the Longitude and Latitude coordinates of the component *i*, while *m*_i_ is the mass of that component. For the population dataset, the mass is considered as the population density per km^2^, while for the dam dataset, the mass is considered as the maximum reservoir capacities. For each dam, reservoir capacity and time of construction were estimated from the GRanD v1.3 dataset (Lehner et al. 2011), while the spatial and temporal distribution of population density in the US was derived from the 1-km decadal maps developed by Fang and Jawitz (2018).

This method has some caveats. First, because of its focus, our large-scale analysis of human population in the US did not engage with a significant part of American history related to Native-American tribes, who lived across North America, and the fact that the westward expansion was far from peaceful (Berlepsch and Rodríguez-Pose 2019). Second, while we focused on the role of dams and water supply, there are other drivers that contribute to explain spatial and temporal changes in the distribution of human population and water demand over the past two centuries. These include, among others, land fertility and potential for agricultural growth in the West. Hence, our large-scale analysis of spatial and temporal trends is primarily descriptive and exploratory. There is no attempt to find causal links.

### Statistical analysis

We carried out a lag-correlation analysis in order to explore supply-demand cycles in the Southwest with a focus on agriculture and irrigation. The analysis was performed for the period between 1950 and 1980, with steps of 5 years, as water use data are only available from the USGU dataset (https://water.usgs.gov/watuse/data/) starting from 1950. Data for water supply, instead, were derived from the GRanD dataset (Lehner et al. 2011) as the total reservoir capacity of dams having irrigation as primary or secondary purpose.

We tested two hypotheses: (i) water supply is a predictor of water use; (ii) water use is a predictor of water supply. To this end, we computed linear correlations between the vector of the water supply and the shifted (lagged) vector of water use, and vice versa. For example, the cross-correlation value with a lag time of −10 years corresponds to the correlation between water use lagged by 10 years and water supply without lag. Lag-time values spacing from −10 years to +10 years with 5 year-interval steps were considered in this study.

## Results

In this section, we first describe the results of our large-scale analysis of spatial and temporal trends in dam development and population growth over the past two centuries in the US. This allows us to identify three main phases with different dominant processes at play. To better explore the interplay of water supply and demand, we then focus on agricultural water use in the Southwest, and examine chicken-and-egg dynamics.

### Spatial and temporal trends

**Fig. 2:**
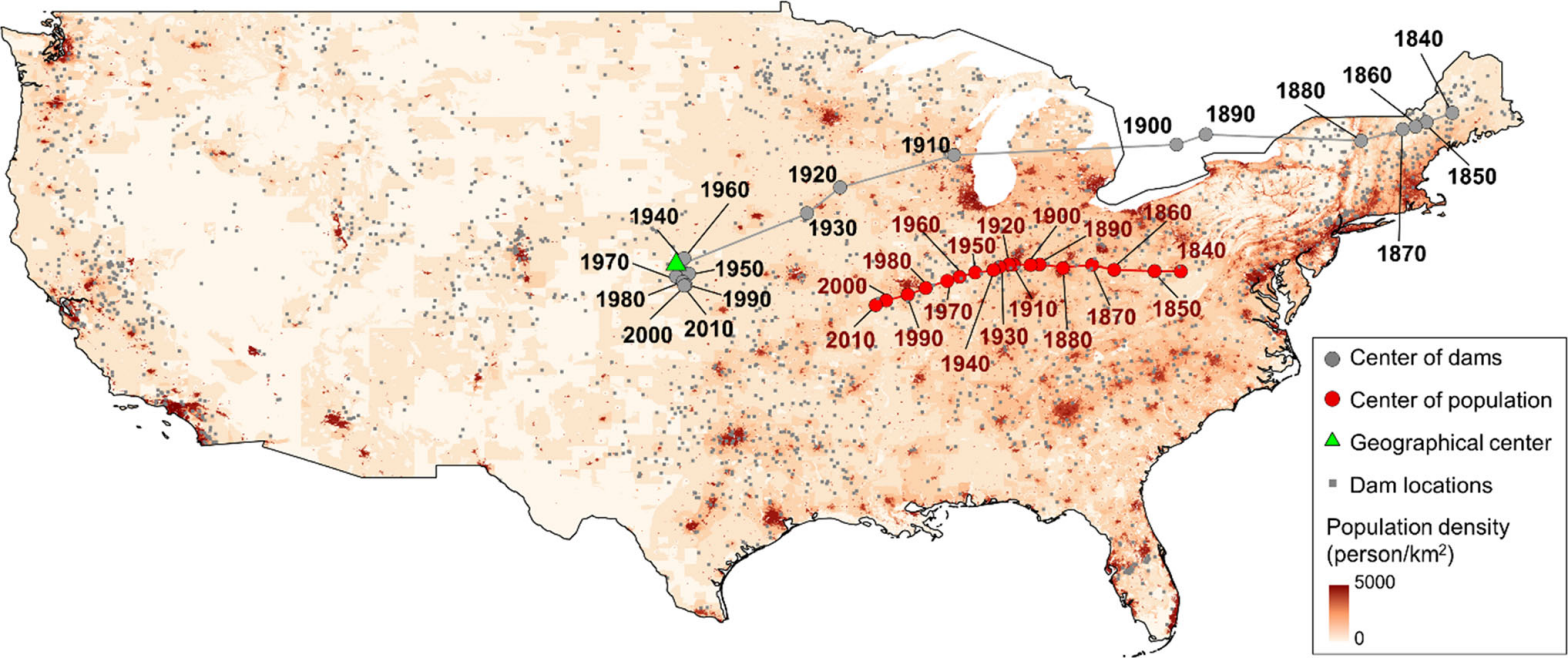
Center of mass (CoM) of human population (red dots) and dams (grey dots) in the period between 1840 and 2010. The map also shows the location of the geographical center of the contiguous US (green triangle), as well as the location of dams (Lehner et al. 2011) and population density (Fang and Jawitz 2018) in the year 2010

To explore large-scale trends, we examined the spatial distribution of dams and human population across the US by mapping changes in time of their CoM (Fig. 2) in the period between 1840 and 2010. Figure 2 shows the results of this macroscopic analysis highlighting the westward expansion of both human population and dams. It can be observed that a rapid westward expansion of dams occurred between 1880 and 1960, while in the subsequent years the CoM of dams tends to fluctuate around the geographical center of the US. On the other hand, a more continuous westward expansion is found for the US population.

The contemporary US are characterized by a marked bicoastal population distribution, with high concentrations in urban areas (Fang and Jawitz 2018), as depicted by the background of Fig. 2. This pattern is the outcome of two interrelated migration processes that took place over two centuries: migration to and within the American continent. The first concerns migrations from the ‘Old Continent’ to the ‘New World’. In the period between the 1850s and World War I, approximately 25 million people migrated from Europe to the American continent (Hatton and Williamson 1992; Bertocchi and Strozzi 2006). This mass migration left a profound imprint on both institutions and economic development of the US that is still felt today (Rodríguez-Pose and von Berlepsch 2014).

### Dam development phases

**Fig. 3:**
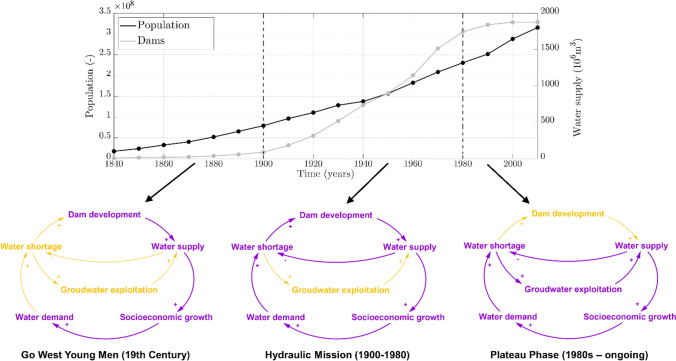
Temporal trends of total population and reservoir capacity in the US. The two curves are used to derive three main phases, whose dominant processes are highlighted in purple in the three loop diagrams

Temporal trends in population growth and reservoir capacity were used to identify three main phases of dam development in the US (Fig. 3), which we term ‘Go West Young Men’, ‘Hydraulic Mission’, and ‘Plateau Phase’.

Moreover, based on the historical background (“Historical background”), we conceptualized the dominant processes characterizing these three phases, as positive (+) and negative (−) feedbacks between dam development, socioeconomic growth, water supply and demand (Fig. 3, bottom panels).

In the ‘*Go West Young Men*’ phase, human population grew faster than reservoir capacity (Fig. 3). In this period, several dams were built for multiple purposes including navigation (“Historical background”), but water was not a limiting factor and increasing water demands could be met without major risks of water shortage. The rapid growth in population was paralleled by high rates of internal geographic mobility (Ferrie 2005), which largely exceeded that of other Western country (Berlepsch and Rodríguez-Pose 2019). Geographical mobility has mostly taken the form of westward migration, with the geographical center of the population constantly shifting towards the West (Fig. 2). Westward expansion meant increased access to land and water resources. This triggered an exponential GDP growth, which increased by 175-folds between the end of the 18th and the beginning of the 20th century (Gallman 2000).

In the ‘*Hydraulic Mission*’ phase, reservoir capacity grew faster than human population (Fig. 3). This was the era of greater dam expansion in the US. Drawing on foundational work of Swyngedouw (1999), Wester (2009), Molle et al. (2009) and Warner et al. (2017), we use the term hydraulic mission to describe a development paradigm grounded on state‐led water resources development through large scale hydraulic infrastructures. Underlying this paradigm is the strong conviction that “every drop of water flowing to the ocean is a waste” (Wester 2009). This triggered and justified the pursuit of iconic hydropower or largescale public irrigation projects (Molle et al. 2009). Whilst contributing to welfare, these projects strategically worked to developing powerful hydraulic bureaucracies, and to controlling and engineering state-space (Scott 1999; Kaika 2006; Molle et al. 2009; Wester 2009: Rusca et al. 2019). The Bureau of Reclamation, established in 1902 by Theodore Roosevelt, played a key role in defining and implementing the ‘hydraulic mission’ of the US. Large dams were built to secure water supply and enable socioeconomic growth, especially in dryer parts of the US (Southwest). The top panel of Fig. 3 shows that reservoir capacity grew faster than human population. This enabled urban, industrial and agricultural expansions and thus higher levels of water use, i.e. supply-demand cycles (Kallis 2010; Gohari et al. 2013; Di Baldassarre et al. 2018).

In the ‘*Plateau phase*’, reservoir capacity has remained essentially stable and dam development was no longer a dominant process (Fig. 3). The end of the hydraulic mission can be partly attributed to increased awareness of the social and environmental impacts of dams and reservoirs, observed over the past decades in many western countries (Vörösmarty et al. 2000; Gleick 2003; AghaKouchak et al. 2015; Veldkamp et al. 2017). Yet, high levels of water demand, which are a legacy of the hydraulic mission, are being met more and more via groundwater exploitation, especially in the Southwest region (Perrone and Jasechko 2019).

### The interplay of water supply and use

To further explore the spatial and temporal coevolution of water supply and water use over the contiguous US, we focused on agriculture water use during hydraulic mission. Data from USGS are only available starting from 1950, so we focused on the period between 1950 and 1980, i.e. end of the hydraulic mission phase.

**Fig. 4:**
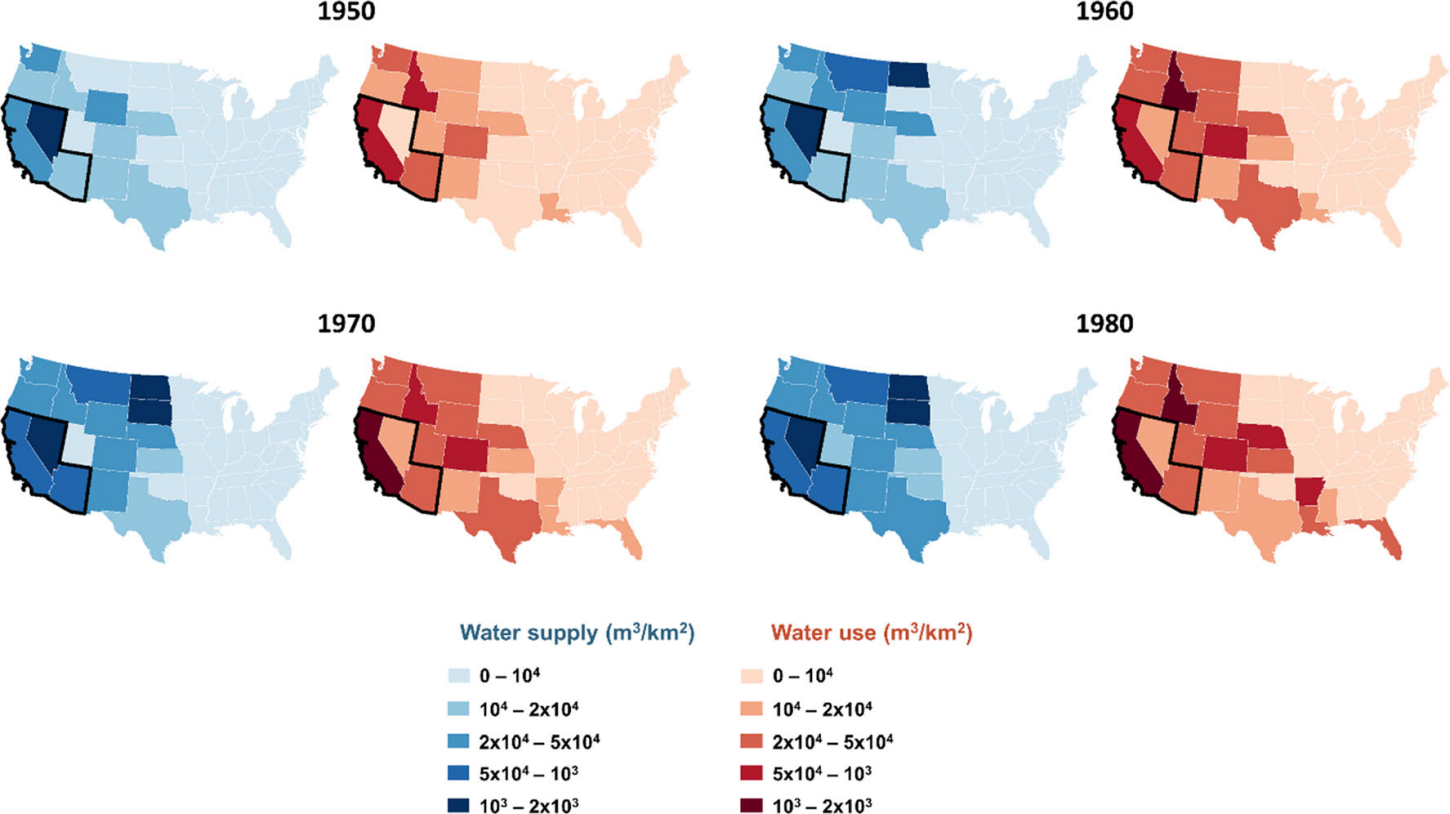
Water supply and use for irrigation. Data for water supply are derived from the GRanD dataset (Lehner et al. 2011) considering only the dams that have irrigation as primary or secondary purpose. Irrigation water use in the US are based on the dataset provided by the USGS and freely available online (https://water.usgs.gov/watuse/data/)

Figure 4 shows the spatial distribution of water supply and use for irrigation across the US between 1950 and 1980, normalized over the area of each US state. In this period, agricultural water use increased exponentially, especially in the Southwest region (Fig. 4).

**Fig. 5:**
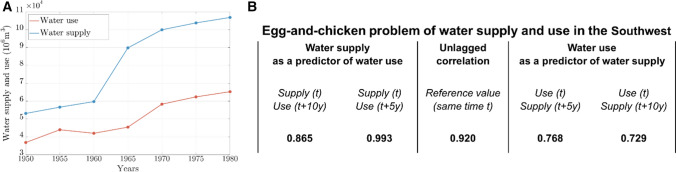
Supply demand cycle in the Southwest. **a** Water supply and use for irrigation purposes. **b** The interplay of water supply and irrigation: lagged-correlation values for different lags at 5 years’ time step

The legacy of dams in the US is particularly evident in the Southwest region. Figure 5a shows that both reservoir capacity increased substantially in the Southwest in the period between 1950 and 1980 (hydraulic mission).

It is often suggested that large water infrastructure in the Southwest was the engineering response to the growing population and agricultural activities in the region. In contrast, Ingram and Malamud-Roam (2015) contend that the massive engineering has actually driven the rapid growth to an extent that is far above what the region can support. To unravel this egg-and-chiken problem of water supply and demand, we performed a lag-correlation analysis of the historical trends of water supply and water use for irrigation in the Southwest region during the hydraulic mission phase. We tested two hypotheses using lagged correlations: (i) water supply is a predictor of water use; (ii) water use is a predictor of water supply. We found that none of these two hypotheses can be rejected as all lagged cross-correlation coefficients (Fig. 5b) are statistically significant (*p* < 0.01). Yet, we found higher correlation coefficients when using water supply as a predictor of water use. For example, the correlation coefficient between water supply at the time *t* and water use at the time *t* + 5 years is 0.993.

We interpret this outcome as a major manifestation of supply-demand cycles in the Southwest region. Increasing water supply also enables increasing water use. While this dynamic can be considered as one facet of economic growth, it has also reinforced spirals towards unsustainable water demands.

High demands from the agricultural section have made the Southwest heavily reliant on water infrastructure. As dam development has plateaued, wells are getting deeper and groundwater levels are declining too (Perrone and Jasechko 2019). Meanwhile, drought exposure has increased because of population growth and agricultural expansion (AghaKouchak et al. 2015; Carrao et al. 2016). This trend is set to worsen in the coming decades because of climate change (Breinl et al. 2020; Williams et al. 2020), with increasingly catastrophic outcomes during droughts (Berbel and Esteban 2019), including severe water shortages (Di Baldassarre et al. 2018) and overexploitation of groundwater resources (Castle et al. 2014).

## Discussion and conclusions

We examined the interplay between water supply and demand with a focus on the role of dam developments. Our large-scale analysis of spatial and temporal trends in the US showed that the coevolution in space and time of people and dams over the past two centuries was characterized by three distinct phases, in which different processes dominated the interplay. The focus on agricultural water use in the Southwest region has shown that there is neither cause nor effect in the interplay, but rather a chicken-and-egg dynamic as water supply partly meets and partly enables water use.

We showed that the US have got into a lock-in condition with unsustainable levels of water consumption. As a result, the agricultural sector is increasingly relying on groundwater while drought conditions lead to severe water crises, especially in the Southwest region. Thus, it is critical that government and agricultural sector embrace a sustainable trajectory in view of water availability and climatic conditions in the region. Moreover, while the US reached a plateau and recognized the importance of reducing heavy reliance on large water infrastructure (Gleick 2003), climate change poses new challenges and policy-makers in the US are currently advocating again for supply-side solutions, including dam augmentation and aquifer storage and recovery (Perry and Praskievicz 2017).

To provide policy insights, we refer to the concept of legacy risks, which was originally proposed in the fields of mining and nuclear waste (Russell 2000; Pepper and Roche 2014), but later applied to a range of processes, including environmental management (Winiwarter et al. 2016). Reducing legacy risks means identifying development trajectories that may result unsustainable for the next generations (Di Baldassarre et al. 2019). In our study, we proposed the identification of legacy risks as one way to examine the sustainability of dam development over a long-time horizon. We showed how the legacy of large dams can generate lock-in conditions that are difficult (if not impossible) to reverse. Our results in the US and, in particular, the Southwest are similar to previous studies in other parts of the world in which large water infrastructure has secured water in otherwise dry areas, including Athens (Kallis 2010), Cape Town (Rodina 2019), and Melbourne (Garcia et al. 2020).

As past decisions were a legacy to the present, current decisions will be a legacy for the future. Hence, the results of our study are also relevant to the current debate about the sustainability of new dams and reservoirs, which are being planned or built in many places around the world (Zarfl et al. 2015; Crow-Miller et al. 2017; Latrubesse et al. 2017; Rusca et al. 2019). Emerging and developing regions of the world are on the verge of starting a trajectory of heavy reliance on large dams, which present similarities to the one described here. Several countries, such as Brazil (Fearnside 2015), are mobilizing resources to quench the thirst of growing cities or increase agricultural production. Our analysis of the legacy of dams in the US provides insights into what type of socio-environmental trajectories might follow these developments.
